# Physiological and Biochemical Mechanisms of Seed Priming-Induced Chilling Tolerance in Rice Cultivars

**DOI:** 10.3389/fpls.2016.00116

**Published:** 2016-02-09

**Authors:** Saddam Hussain, Fahad Khan, Hafiz A. Hussain, Lixiao Nie

**Affiliations:** ^1^National Key Laboratory of Crop Genetic Improvement, MOA Key Laboratory of Crop Ecophysiology and Farming System in the Middle Reaches of the Yangtze River, College of Plant Science and Technology, Huazhong Agricultural UniversityWuhan, China; ^2^College of Resources and Environment, Huazhong Agricultural UniversityWuhan, China; ^3^Department of Agronomy, University of AgricultureFaisalabad, Pakistan; ^4^Hubei Collaborative Innovation Center for Grain Industry, Yangtze UniversityJingzhou, China

**Keywords:** antioxidants, chilling, germination, oxidative stress, respiration, starch metabolism

## Abstract

Rice belongs to tropical and subtropical environments and is extremely sensitive to chilling stress particularly during emergence and early stages of seedling development. Seed priming can be a good approach to enhance rice germination and stand establishment under chilling stress. The present study examined the role of different seed priming techniques viz., hydropriming, osmopriming, redox priming, chemical priming, and hormonal priming, in enhancing the chilling tolerance in rice. The most effective reagents and their pre-optimized concentrations based on preliminary experiments were used in this study. Two different rice cultivars were sown under chilling stress (18°C) and normal temperatures (28°C) in separate growth chambers. A non-primed control treatment was also maintained for comparison. Chilling stress caused erratic and delayed germination, poor seedling growth, reduced starch metabolism, and lower respiration rate, while higher lipid peroxidation and hydrogen peroxide accumulation in rice seedlings of both cultivars. Nevertheless, all the seed priming treatments effectively alleviated the negative effects of chilling stress. In addition, seed priming treatments triggered the activities of superoxide dismutase, peroxidase, and catalase, and enhanced the accumulations of glutathione and free proline in rice seedlings, which suggests that these measures help prevent the rice seedlings from chilling induced oxidative stress. Chemical priming with selenium and hormonal priming with salicylic acid remained more effective treatments for both rice cultivars under chilling stress than all other priming treatments. The better performance and greater tolerance of primed rice seedlings was associated with enhanced starch metabolism, high respiration rate, lower lipid peroxidation, and strong antioxidative defense system under chilling stress.

## Introduction

Low temperature is one of the major factors limiting the productivity and the geographical distribution of many important field crops. Rice, a staple food for more than half of the world’s population, belongs to tropical and subtropical environments with an optimum growth temperature around 28°C (Kovach et al., 2007; Sang and Ge, 2007). Most of the rice cultivars are extremely sensitive to chilling stress particularly during emergence and early stages of seedling development. Yoshida (1981) reported that the speed and percentage of germination of rice seeds can be decreased if the daily mean temperature was below 20°C. Worldwide, chilling stress during rice growing season constitutes a serious problem in many countries including Australia, China, Indonesia, Japan, South Korea, Nepal, the United States (Yoshida, 1981), Southern Latin America mainly Chile (Castillo and Alvarado, 2002), and Brazil (Cruz and Milach, 2004). Moreover in China, double direct-seeded rice system has attracted the interest of many researchers and growers due to less irrigation water and labor demands, along with higher resource use efficiency (Xu et al., 1995; Peng, 2014). Double direct-seeded rice system is the process of crop establishment by directly sowing the dry/soaked seeds in the field in both early and late seasons rather than nursery transplanting. Nevertheless, direct-seeded early rice (usually sown in spring-early April) in this system suffers chilling stress at germination and early seedling growth stages, which raises the risk of huge grain yield loss.

Chilling stress thermodynamically depresses the kinetics of many physiological and metabolic processes in plants (Ruelland et al., 2009). It has been widely reported that chilling stress severely hampers the uniformity and rate of germination, seedling vigor, and delays the plant development stages (Kang and Saltveit, 2002; Cruz and Milach, 2004; Cheng et al., 2007; Oliver et al., 2007), leading to severe yield losses (Oliver et al., 2007; Ruelland et al., 2009). The deleterious effects of chilling stress on crop plants presumably arise due to the induction of oxidative stress. Chilling stress generates a large amount of reactive oxygen species (ROS) in plants cell and triggers lipid peroxidation in membranes (Guo et al., 2006; Gill and Tuteja, 2010; Shu et al., 2011). The excessive production of ROS is highly toxic to cellular and metabolic functions of the plants and causes damage to proteins, lipids, carbohydrates and DNA, which ultimately results in cell death (Gill and Tuteja, 2010). The perturbation of chloroplastic and mitochondrial metabolism under excessive ROS generations also leads to reduced respiration rate and energy supply for growing plant tissue (Taylor et al., 2002).

To overcome these conditions, plants have evolved the antioxidant defense systems including antioxidant enzymes (e.g., superoxide dismutase, SOD; catalase, CAT; peroxidase, POD) and non-enzymatic antioxidants (e.g., ascorbic acid, AsA; glutathione, GSH) which may neutralize or counteract the harmful effects of ROS and protect the plants from oxidative stress (Foyer and Noctor, 2005; Gill and Tuteja, 2010; Anjum et al., 2015; Chen et al., 2015). Previously, it has been found that the activities of antioxidants under chilling stress were correlated with stress tolerance and chilling-tolerant maize (Hodges et al., 1997; Taka, 2004), cucumber (Kang and Saltveit, 2002), and rice (Kang and Saltveit, 2002; Huang and Guo, 2005) cultivars had higher anti-oxidation activities. Moreover, carbohydrate metabolism and synthesis of metabolites like proline and soluble sugars have also been reported to be involved in chilling tolerance of plant species (Hayat et al., 2012; Nägele et al., 2012).

In recent years, various strategies are being employed in order to induce the abiotic stress tolerance in plants. Seed priming is an effective, practical and facile technique to enhance rapid and uniform emergence, high seedling vigor, and better yields in many field crops particularly under unfavorable environmental conditions (Jisha et al., 2013; Paparella et al., 2015). Seed priming is a controlled hydration technique that triggers the normal metabolic processes during early phase of germination before radicle protrusion (Hussain et al., 2015). Higher and synchronized germination of primed seeds primarily occurs due to reduction in the lag time of imbibition (Brocklehurst and Dearman, 2008), enzyme activation (Lee and Kim, 2000), build-up of germination enhancing metabolites (Hussain et al., 2015), metabolic repair during imbibition (Farooq et al., 2006), and osmotic adjustment (Bradford, 1986). Various seed priming techniques including hydropriming, osmopriming, chemical priming, nutrient priming, hormonal-priming, and redox priming are employed in rice under wide range of environmental stresses (Jisha et al., 2013; Paparella et al., 2015). There has been increasing evidence that primed plants exhibit activation of cellular defense responses, which imparts tolerance to subsequent exposure to biotic or abiotic stresses in the field (Jisha et al., 2013). Recently, various studies have reported the beneficial effects of seed priming under chilling stress in different crops. For instance, Xu et al. (2011) found that the seed priming improved the chilling tolerance in tobacco during seed germination and seedling growth by the activation of antioxidant system in the plant tissues. Guan et al. (2009) reported that seed priming enhanced the speed of maize seed germination and triggered the seedling growth under chilling stress. Likewise, Elkoca et al. (2007) recommended the hydropriming and osmopriming for a better germination and vigorous growth of chickpeas under low-temperature conditions.

Despite the dearth of literature on the useful effects of seed priming under abiotic stresses (Jisha et al., 2013), little work has been done regarding the comparative performance of different seed priming techniques in enhancing the chilling tolerance of rice and to explore the mechanisms of priming-induced stress tolerance. Therefore, the present study examined the various seed priming techniques viz., hydropriming, osmopriming, redox priming, chemical priming, and hormonal-priming against chilling stress in rice. The effective reagents were selected and their concentrations were pre-optimized in various preliminary studies based on rice germination and seedling growth performance (Hussain et al., unpublished). In addition, the present study also unraveled the physiological and biochemical changes in rice seedlings under the influence of seed priming and chilling stress to obtain a better understanding of priming-induced mechanisms and to provide a basis for further analyses.

## Materials and Methods

### Plant Material

Seeds of two widely grown *Indica* rice cultivars, Huanghuazhan (HHZ, inbred) and Yangliangyou6 (YLY6, hybrid) with initial germination of >95% and initial seed moisture content of below 10% (on dry weight basis) were used in these studies. To minimize contamination during priming, seeds were surface sterilized with 2.63% NaOCl solution (household bleach diluted 1:1 with sterile water) for 30 min and rinsed three times with sterile distilled water.

### Experimental Details

In order to ascertain the role of seed priming in alleviating the adverse effects of chilling stress, different seed priming approaches viz., osmopriming (CaCl_2_: 100 mg L^-1^ calcium chloride), redox priming (H_2_O_2_: 50 μM hydrogen peroxide), chemical priming (Se: 50 μM selenium), and hormonal priming (SA: 100 mg L^-1^ salicylic acid) were examined. The effective levels of these priming reagents were pre-optimized based on rice emergence and early seedling growth performance. Treatments selected for this study were, [1] no priming + control temperature (NP + Cn), [2] no priming + chilling stress (NP + CS), [3] hydropriming + chilling stress (HP + CS), [4] CaCl_2_ priming + chilling stress (CaCl_2_ + CS), [5] H_2_O_2_ priming + chilling stress (H_2_O_2_ + CS), [6] Se priming + chilling stress (Se + CS), and [7] SA priming + chilling stress (SA + CS). Seeds were primed in the dark at 25°C for 24 h with constant gentle agitation. The ratio of seed weight to solution volume (w/v) was 1:5. The priming solution was changed every 12 h. Autoclaved distilled water was used for hydropriming. After 24 h, the primed seeds were washed with distilled water for 2 min, surface-dried using blotting paper, and transferred to an air-drying oven at 25°C for 48 h to reduce the moisture content to <10%. The chilling stress was imposed in growth chamber by maintaining the day and night temperatures at 18°C, while temperature (day/night) for control treatment was set at 28°C in a separate growth chamber. In both growth chambers, a 12-h light period and humidity of 60% were maintained throughout the study.

Forty healthy seeds from each treatment were evenly germinated on two layers of filter paper in 14.5 cm diameter Petri dishes. After adding 20 ml water to each replicate, Petri dishes were covered with lid and placed on steel racks in growth chamber. Equal volume of distilled water was applied to all Petri dishes when their moisture content declined. All the treatments were laid out in a completely randomized design replicated six times and were repeated thrice for recording physiological and biochemical attributes.

### Observations

#### Germination and Seedling Growth

Germination of seeds was recorded on daily basis according to AOSA (1990) till it became constant. Seed was considered to be germinated when radicle length exceeded 2 mm. Shoot and root length of 10 randomly selected seedlings from each replication were measured at 10 days after sowing (DAS). Seedlings of each replicate were dissected into roots and shoots and their fresh weight was recorded immediately.

#### Starch Metabolism

Starch metabolism in rice seedlings were recorded at 1, 3, 5, and 7 DAS. For determination of α-amylase activity, 1.0 g fresh seedling sample was ground and mixed with 100 ml distilled water, and left for 24 h at 4°C. The enzyme activity was determined from supernatant liquid by dinitrosalicylic acid (DNS) method (Bernfeld, 1955; Lee and Kim, 2000). To determine total soluble sugars, ground seedling sample (1 g) was mixed with 10 mL distilled water and left for 24 h at 25°C. Mixture was filtered with Whatman No. 42 (Whatman plc, Kent, UK) and the final volume was made to 10 ml with distilled water. Total soluble sugars were determined by the phenol sulphuric method (DuBois et al., 1956).

#### Respiration Rate

Respiration rates were determined using small-skep-method (Li et al., 2015) at 1, 3, 5, and 7 DAS. Briefly, 5 g of rice seedlings were sampled from each replicate and were immediately put into a 0.5 L of glass bottle, which was connected to a close-circuit system. The CO_2_ concentration in the bottle was recorded after every minute. Respiration rate was calculated based on the increase in CO_2_ concentrations within 1 min.

#### Free Proline Content

Free proline content in rice seedlings was determined through Bates et al. (1973) method. Briefly, harvested samples were homogenized in 5 mL of 3% sulfo-salicylic acid and centrifuged at 6,000 rpm for 10 min. Supernatant (2 mL) was heated with 2 mL of ninhydrin and 2 mL glacial acetic acid at 100°C for 1 h. The reaction mixture was further extracted with 4 mL of toluene by vigorously vortexing for 30 s. The absorption of chromophore was determined at 520 nm (Tecan-infinite M200, Switzerland). The concentration of proline in the samples was estimated by referring to a standard curve of L-proline.

#### Lipid Peroxidation and H_2_O_2_ Content

Lipid peroxidation in rice seedlings was determined as malondialdehyde (MDA) content using thiobarbituric acid method (Bailly et al., 1996) while the level of H_2_O_2_ was measured according to the method described by Patterson et al. (1984). The MDA as well as H_2_O_2_ contents were expressed as μmol g^-1^ FW.

#### Antioxidant Enzymes Activities and GSH Content

The activities of enzymatic antioxidants viz., SOD, POD, and CAT, were determined according to the methods given in Zheng et al. (2015). The SOD activity was determined by using “SOD Detection Kit” (A001-1) purchased from Nanjing Jiancheng Bioengineering Institute and was presented as U g^-1^ FW. One unit of SOD activity was the amount of extract that gives 50% inhibition in reduction of xanthine as monitored at 550 nm (Tecan-infinite M200, Switzerland). The POD activity was based on the determination of guaiacol oxidation at 470 nm by H_2_O_2_ and was expressed as U g^-1^ FW. The change in absorbance at 470 nm was recorded for every 20 s by spectrophotometer. One unit of POD activity is the amount of enzyme that will cause the decomposition of 1 μg substrate at 470 nm (HITACHI U-3900) for 1 min in 1 g fresh sample at 37°C. The CAT activity was measured using CAT detection kit (A007-1, Nanjing Jiancheng Bioengineering Institute), and was demonstrated as U g^-1^ FW. One unit of CAT activity was defined as the amount of enzyme that will cause the decomposition of 1 μmol H_2_O_2_ at 405 nm (Tecan-infinite M200, Switzerland) per second in 1 g fresh sample at 37°C. The GSH content was determined using “Glutathione Assay Kit” (A006, Nanjing Jiancheng Bioengineering Institute) for reduced glutathione, which followed the DTNB [5,5’-dithiobis (2-nitrobenzoic acid)] method. The absorbance measured at 420 nm (Tecan-infinite M200, Switzerland) and GSH content was expressed as μmol g^∘1^ protein.

### Statistical Analysis

All the data recorded from the two rice cultivars are presented as the mean ± standard error (SE) of six replicates. Analyses were performed using the software Statistix 9.0, and the mean variance of the data was analyzed using the least significant difference (LSD) test at the 0.05 probability level.

## Results

### Seed Priming Enhanced the Speed and Rate of Rice Germination Under Chilling Stress

**FIGURE 1:**
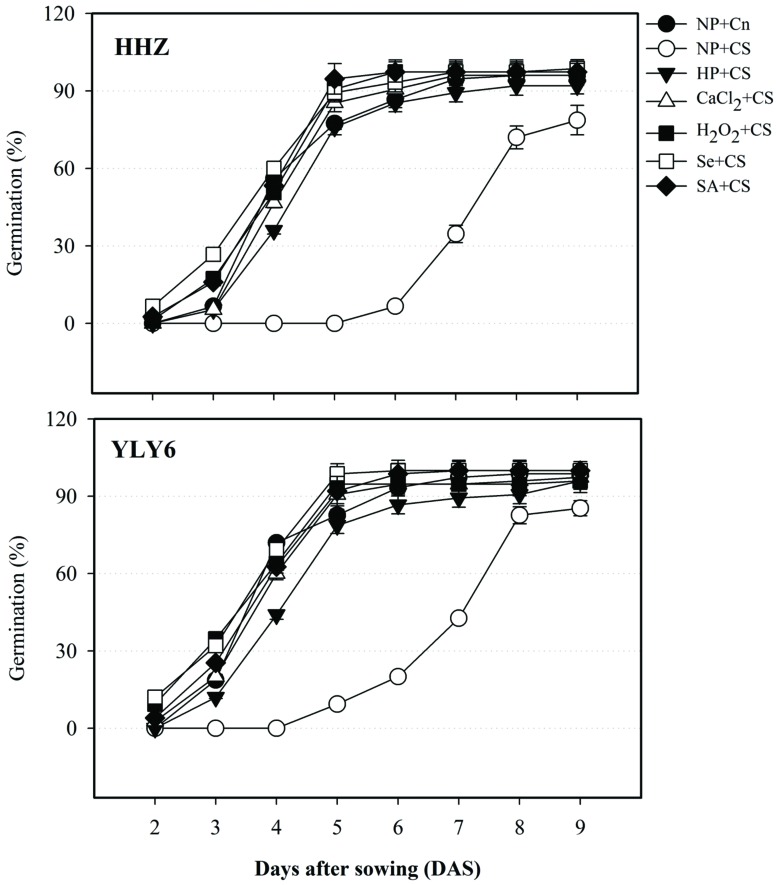
**Influence of various seed priming treatments on germination dynamics of two rice cultivars under chilling stress.** Vertical bars above mean indicate standard error of six replicates. NP + Cn: no priming and no stress, NP + CS: no priming and chilling stress, HP + CS: hydropriming and chilling stress, CaCl_2_ + CS: calcium chloride priming and chilling stress, H_2_O_2_ + CS: hydrogen peroxide priming and chilling stress. Se + CS: selenium priming and chilling stress, and SA + CS: salicylic acid priming and chilling stress, Cn: 28°C, CS: 18°C, HHZ: inbred cultivar huanghuazhan, YLY6: hybrid variety Yangliangyou-6.

Data regarding germination dynamics of primed and non-primed seeds of two rice cultivars under chilling stress are shown in **Figure 1.** Exposure of chilling stress in non-primed seeds (NP + CS) considerably decreased the speed and rate of rice germination; therefore, none of the seed in NP + CS could germinate in both cultivars at 4 DAS, compared with >50% germination in NP + Cn. The rice cultivars HHZ and YLY6, took 6 and 5 days to start germination in NP + CS treatment, respectively (**Figure 1**). All the seed priming treatments were effective to alleviate the chilling-induced delay of germination. At 4 DAS, 40, 53, 57, 64, and 58% seeds were found to be germinated in HP + CS, CaCl_2_ + CS, H_2_O_2_ + CS, Se + CS, and SA + CS treatments, respectively, averaged across two cultivars. Rice hybrid cultivar YLY6 depicted faster germination under chilling stress compared with inbred cultivar HHZ. Evaluation of final germination percentage (9 DAS) depicted that germination of HHZ and YLY6 in NP + CS were significantly reduced by 18 and 13%, respectively, compared with NP + Cn. Nevertheless, all the priming treatments recorded >92% germination at 9 DAS in both rice cultivars. The final germination of rice cultivars in CaCl_2_ + CS, H_2_O_2_ + CS, Se + CS, and SA + CS treatment was statistically similar with that in NP + Cn (**Figure 1**).

### Seed Priming Alleviated the Chilling-Induced Inhibition of Rice Seedling Growth

**FIGURE 2:**
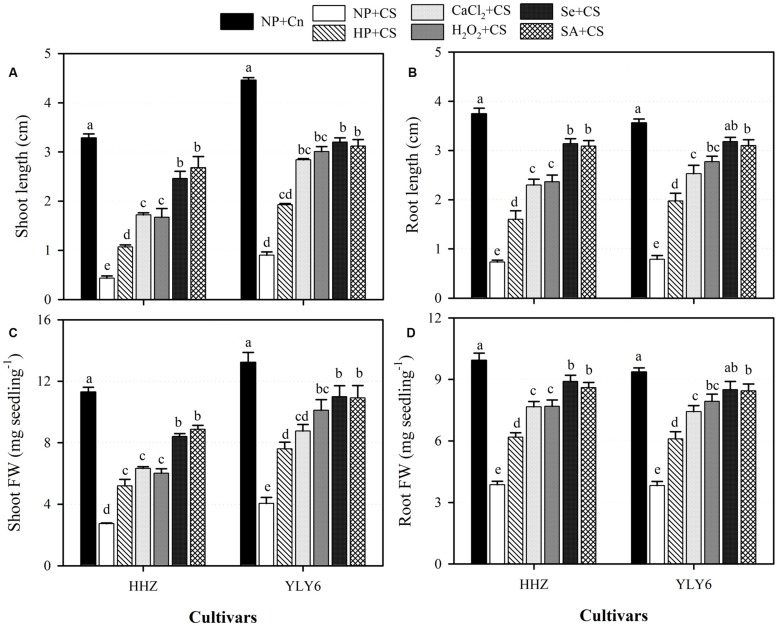
**Influence of various seed priming treatments on **(A)** shoot length, **(B)** root length, **(C)** shoot fresh weight, and **(D)** root fresh weight of two rice cultivars under chilling conditions.** Vertical bars above mean indicate standard error of six replicates. Mean value for each treatment with different lowercase letters indicate significant differences by the LSD-test (*P* < 0.05). NP + Cn: no priming and no stress, NP + CS: no priming and chilling stress, HP + CS: hydropriming and chilling stress, CaCl_2_ + CS: calcium chloride priming and chilling stress, H_2_O_2_ + CS: hydrogen peroxide priming and chilling stress. Se + CS: selenium priming and chilling stress, and SA + CS: salicylic acid priming and chilling stress, Cn: 28°C, CS: 18°C, HHZ: inbred cultivar huanghuazhan, YLY6: hybrid variety Yangliangyou-6.

Chilling stress was found to severely hamper the seedling growth of both rice cultivars (**Figure 2**). Compared with NP + Cn, the shoot length, root length, shoot fresh weight, and root fresh weight of NP + CS were decreased by 83, 79, 72, and 60%, respectively, across cultivars. Nevertheless, seed priming treatments were effective in alleviating the chilling-induced inhibition of growth. When compared with NP + CS, different seed priming treatments significantly increased the shoot length of rice cultivars by 56–78%, root length by 57–76%, shoot fresh weight by 47–66%, and root fresh weight by 37–58% (**Figure 2**). When the different priming treatments were evaluated under chilling stress, Se + CS and SA + CS outperformed the other priming treatments, and these two treatments were statistically similar (*P* ≤ 0.05) with each other for all growth attributes in both rice cultivars. Although, the HP + CS was the least effective treatment; it recorded significantly higher growth in both rice cultivars compared with NP + CS, and was statistically similar with CaCl_2_ + CS for shoot fresh weight (**Figure 2**).

### Seed Priming Regulated the Starch Metabolism in Rice Seedlings Under Chilling Stress

**FIGURE 3:**
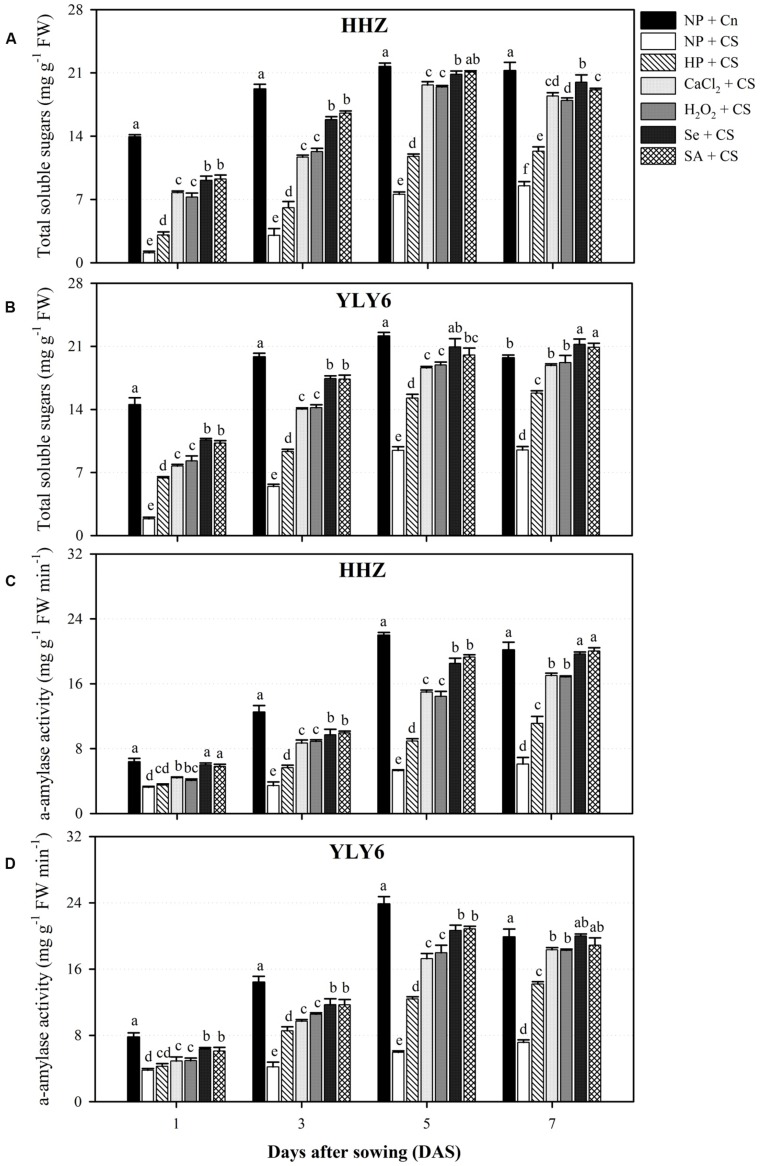
**Influence of various seed priming treatments and chilling stress on starch metabolism at 1, 3, 5, and 7 DAS in two cultivars.** **(A)** total soluble sugars in HHZ, **(B)** total soluble sugars in YLY6, **(C)** α-amylase activity in HHZ, and **(D)** α-amylase activity in YLY6. Vertical bars above mean indicate standard error of six replicates. Mean value for each treatment with different lowercase letters indicate significant differences by the LSD-test (*P* < 0.05). NP + Cn: no priming and no stress, NP + CS: no priming and chilling stress, HP + CS: hydropriming and chilling stress, CaCl_2_ + CS: calcium chloride priming and chilling stress, H_2_O_2_ + CS: hydrogen peroxide priming and chilling stress. Se + CS: selenium priming and chilling stress, and SA + CS: salicylic acid priming and chilling stress, Cn: 28°C, CS: 18°C, HHZ: inbred cultivar huanghuazhan, YLY6: hybrid variety Yangliangyou-6.

Starch metabolism in rice seedlings was assessed in terms of total soluble sugar content and α-amylase activity (**Figure 3**). Temporal data at 1, 3, 5, and 7 DAS regarding total soluble sugar content and α-amylase activity of both rice cultivars revealed significant (*P* ≤ 0.05) variations under the influence of chilling stress and seed priming treatments. Total soluble sugar content as well as α-amylase activity in both rice cultivars was progressively increased from 1 to 5 DAS in all the treatments (**Figure 3**). Compared with NP + Cn, the total soluble sugar content and α-amylase activity of rice seedlings in NP + CS were reduced by 89 and 50% at 1 DAS, 78 and 72% at 3 DAS, 61 and 75% at 5 DAS, and 56 and 67% at 7 DAS, respectively, across cultivars. The total soluble sugar content and α-amylase activity of rice seedlings starting from 1 to 9 DAS were significantly higher in all the seed priming treatments compared with NP + CS, expect α-amylase activity in HP + CS at 1 DAS (**Figure 3**). Regardless of cultivars, total soluble sugar content at 9 DAS were increased by 56, 108, 107, 129, and 122% in HP + CS, CaCl_2_ + CS, H_2_O_2_ + CS, Se + CS, and SA + CS treatments, respectively, compared with NP + CS. The increases in α-amylase activity for the respective treatments at 9 DAS were 90, 167, 165, 197, and 196%. The Se + CS and SA + CS were the most effective treatments for enhancing starch metabolism in rice seedlings under chilling stress, and these two treatments were statistically (*P* ≤ 0.05) similar with each other for both attributes in both rice cultivars except for total soluble sugar content in HHZ at 9 DAS (**Figure 3**).

### Seed Priming Circumvented the Chilling-Induced Effects on Respiration Rate

**FIGURE 4:**
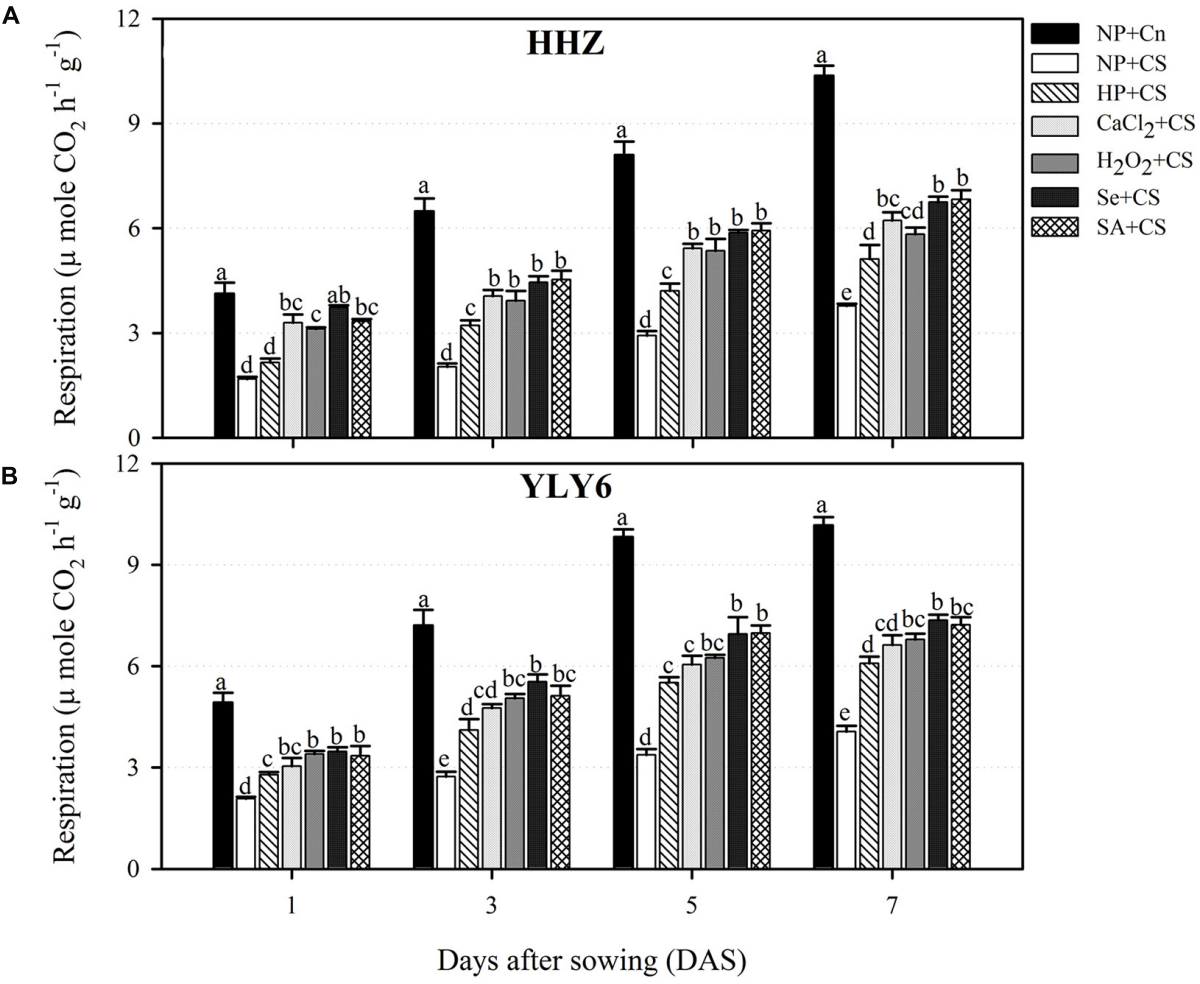
**Influence of various seed priming treatments and chilling stress on respiration rate of rice seedlings at 1, 3, 5, and 7 DAS in two cultivars, **(A)** HHZ, and **(B)** YLY6.** Vertical bars above mean indicate standard error of six replicates. Mean value for each treatment with different lowercase letters indicate significant differences by the LSD-test (*P* < 0.05). NP + Cn: no priming and no stress, NP + CS: no priming and chilling stress, HP + CS: hydropriming and chilling stress, CaCl_2_ + CS: calcium chloride priming and chilling stress, H_2_O_2_ + CS: hydrogen peroxide priming and chilling stress. Se + CS: selenium priming and chilling stress, and SA + CS: salicylic acid priming and chilling stress, Cn: 28°C, CS: 18°C, HHZ: inbred cultivar huanghuazhan, YLY6: hybrid variety Yangliangyou-6.

Temporal data regarding respiration rate of rice cultivars (1, 3, 5, and 7 DAS) under the influence of chilling stress and seed priming treatments are presented in **Figure 4.** Rate of respiration was progressively increased in both rice cultivars achieving the highest values at 7 DAS. Chilling stress was found to hamper the rate of respiration in the rice seedlings of both cultivars. When compared with NP + Cn, the respiration rates in NP + CS at 1, 3, 5, and 7 DAS were decreased by 58, 66, 65, and 62%, respectively, across cultivars (**Figure 4**). Nevertheless, seed priming was effective to circumvent the chilling-induced reduction of respiration rate. In both rice cultivars, all the seed priming treatments recorded significantly (*P* ≤ 0.05) higher respiration compared with NP + CS expect for HP + CS in HHZ at 1 DAS. Under chilling stress, the Se + CS and SA + CS were the most effective treatments regarding respiration rate, however, these two treatments were statistically similar with CaCl_2_ + CS and H_2_O_2_ + CS at 3 and 5 DAS in HHZ, and at 1 DAS in YLY6 (**Figure 4**).

### Seed Priming Increased the Accumulation of Free Proline Under Chilling Stress

**FIGURE 5:**
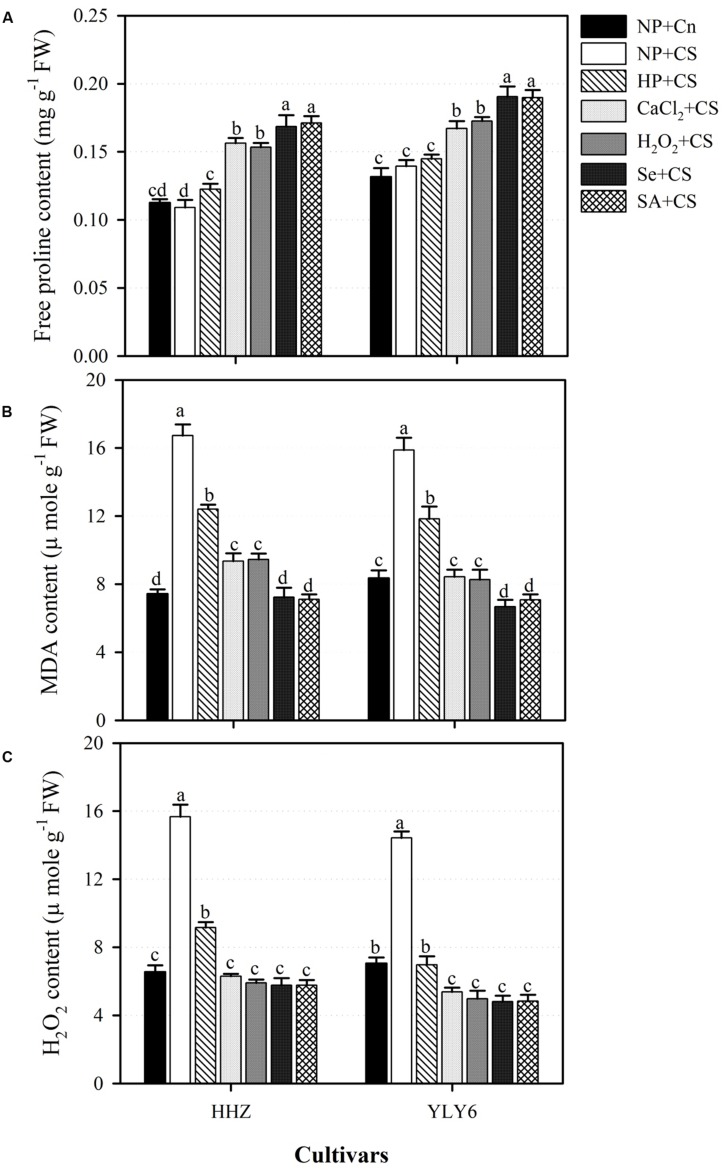
**Influence of various seed priming treatments on **(A)** free proline, **(B)** MDA content, and **(C)** H_2_O_2_ content of rice seedlings under chilling stress.** Vertical bars above mean indicate standard error of six replicates. Mean value for each treatment with different lowercase letters indicate significant differences by the LSD-test (*P* < 0.05). NP + Cn: no priming and no stress, NP + CS: no priming and chilling stress, HP + CS: hydropriming and chilling stress, CaCl_2_ + CS: calcium chloride priming and chilling stress, H_2_O_2_ + CS: hydrogen peroxide priming and chilling stress. Se + CS: selenium priming and chilling stress, and SA + CS: salicylic acid priming and chilling stress, Cn: 28°C, CS: 18°C, HHZ: inbred cultivar huanghuazhan, YLY6: hybrid variety Yangliangyou-6.

Compared with NP + Cn, accumulation of free proline in the seedlings of both rice cultivars was unaffected by NP + CS (**Figure 5A**). Nonetheless, all the seed priming treatments expect HP + CS significantly increased the accumulation of free proline in rice seedlings with respect to NP + CS (**Figure 5A**). Averaged across two rice cultivars, the proline content in CaCl_2_ + CS, H_2_O_2_ + CS, Se + CS, and SA + CS treatments were increased by 23, 24, 31, and 32% compared with NP + CS. The Se + CS and SA + CS were the most effective treatments regarding proline accumulation, and were similar (*P* ≤ 0.05) with each other in both rice cultivars (**Figure 5A**).

### Seed Priming Maintained the Membrane Integrity by Moderating the Chilling-Induced Increases in MDA and H_2_O_2_ Content

Exposure of chilling stress to non-primed rice seedlings (NP + CS) significantly increased the MDA (108%) and H_2_O_2_ (122%) content in the seedlings of both rice cultivars compared with NP + Cn (**Figures 5B,C**). However, all the seed priming treatments significantly assuaged the damaging effects of chilling stress. Compared with NP + CS, the MDA content in HP + CS, CaCl_2_ + CS, H_2_O_2_ + CS, Se + CS, and SA + CS treatments were reduced by 26, 46, 46, 57, and 56%, respectively, averaged across two cultivars (**Figure 5B**). The respective reductions for H_2_O_2_ content were 47, 61, 63, 64, and 64%. All the seed priming treatments except HP + CS were statically similar with each other regarding H_2_O_2_ content, and recorded similar or lower H_2_O_2_ content compared with NP + Cn (**Figure 5C**). However, Se + CS and SA + CS were more effective in reducing MDA content than all other seed priming treatments (**Figure 5B**).

### Seed Priming Triggered the Activities of Antioxidant Enzymes and Enhanced GSH Content Under Chilling Stress

**FIGURE 6:**
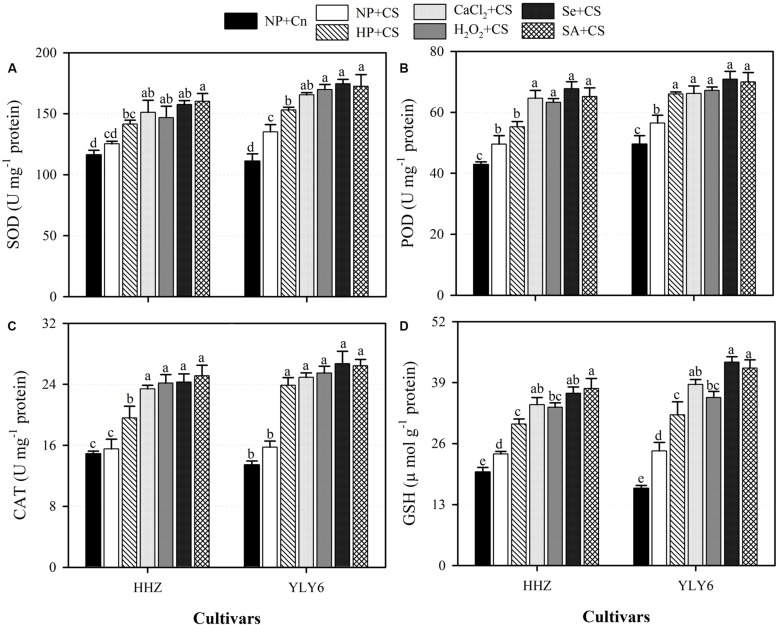
**Influence of various seed priming treatments on activities of enzymatic antioxidants, **(A)** SOD, **(B)** POD, **(C)** CAT, and **(D)** GSH content in rice seedlings under chilling stress.** Vertical bars above mean indicate standard error of six replicates. Mean value for each treatment with different lowercase letters indicate significant differences by the LSD-test (*P* < 0.05). NP + Cn: no priming and no stress, NP + CS: no priming and chilling stress, HP + CS: hydropriming and chilling stress, CaCl_2_ + CS: calcium chloride priming and chilling stress, H_2_O_2_ + CS: hydrogen peroxide priming and chilling stress. Se + CS: selenium priming and chilling stress, and SA + CS: salicylic acid priming and chilling stress, Cn: 28°C, CS: 18°C, HHZ: inbred cultivar huanghuazhan, YLY6: hybrid variety Yangliangyou-6. One unit (U) of SOD activity was defined as the amount of enzyme inhibiting the photochemical reduction of nitro blue tetrazolium chloride by 50% per minute at 560 nm. One unit of CAT was defined as the amount of enzyme required to oxidize 1 μmol H_2_O_2_ min^-1^. One unit of POD activity was defined as the amount of enzyme required to oxidize 1 μmol guaiacol min^-1^.

Data regarding the activities of antioxidant enzymes (SOD, POD, and CAT) and GSH content in rice seedlings under the influence of chilling stress and seed priming treatments are presented in **Figure 6.** Compared with NP + Cn, activities of SOD in HHZ and CAT in both rice cultivars were unaffected by chilling stress (NP + CS), however, POD activity and GSH content in NP + CS treatment were significantly increased in both rice cultivars (**Figure 6**). Under chilling stress, all the seed priming treatments significantly enhanced the activities of antioxidant enzymes as well as GSH content in both rice cultivars compared NP + CS, expect for SOD and POD activities in HP + CS for HHZ cultivar (**Figure 6**). In both rice cultivars, the CaCl_2_ + CS, H_2_O_2_ + CS, Se + CS, and SA + CS treatments were statistically similar (*P* ≤ 0.05) with each other regarding these antioxidants, except H_2_O_2_ + CS was less effective for GSH (**Figure 6**).

## Discussion

Chilling is one of the major abiotic stresses limiting growth and productivity of many field crops. Rice is cultivated in tropical and subtropical environments, and is extremely sensitive to chilling stress particularly at emergence and early stages of seedling development. The present study demonstrated the comparative performance of various seed priming techniques for imparting chilling tolerance in rice at germination and early seedling growth stages. Moreover, the physiological and biochemical basis of seed priming-induced chilling tolerance was examined and discussed.

In the present study, chilling stress was found to severely reduce the speed and success of rice germination in both cultivars. Under chilling stress (NP + CS), germination of rice cultivars was delayed by ≥3 days compared with that in normal (NP + Cn) temperature (**Figure 1**). Previously, several studies have documented the delayed and non-uniform germination of rice under chilling stress (Kang and Saltveit, 2002; Cruz and Milach, 2004; Cheng et al., 2007; Oliver et al., 2007; Ye et al., 2009). Poor and erratic germination under low temperature might be attributed to reduced water uptake (Li et al., 2013), membrane injury, less cellular respiration (Xing and Rajashekar, 2001), and elevated ROS levels (Nayyar et al., 2005). Negative effects of chilling on rice seedling growth were apparent in the form of reduced elongation of roots and shoots, as well as less biomass accumulation (**Figure 2**). Under low temperature, disturbance in seedling morphology is a secondary expression of chilling-induced damage to cell organelles and its interference with key physiological processes. Chilling stress might have reduced the seedlings growth by suppressing cell elongation and division or/and metabolic imbalance in plant tissues (Oliver et al., 2007; Ruelland et al., 2009; Li et al., 2013).

All the seed priming treatments were effective in enhancing the chilling tolerance of both rice cultivars, therefore, rapid and better germination as well as seedling growth of rice were observed in primed-seedlings compared with NP + CS (**Figures 1** and **2**). Better ability of primed rice seeds to complete the germination process in a short time and cope with low temperature conditions might be attributed to readily available substance for germinating seedlings. Farooq et al. (2006) stated that the modulation of hydrolases during lag phase of germination by seed priming helped to build germination metabolites, resulting in earlier and uniform stand establishment. Previously, various studies on maize (Guan et al., 2009), sunflower (Draganić and Lekić, 2012), tobacco (Xu et al., 2011), carrot (Pereira et al., 2009), and chickpea (Elkoca et al., 2007) have reported the enhanced germination and seedling growth under chilling stress.

Starch metabolism during germination and early seedling growth plays a key role in reflecting the seedling vigor particularly under stress conditions. Temporal data of total soluble sugar content and α-amylase activity in rice seedlings revealed that chilling stress was detrimental for both of these attributes, nonetheless, seed priming effectively assuaged the damaging effects of chilling stress (**Figure 3**). Germination and seedling growth of rice under chilling stress were found to be highly associated with the starch metabolism (**Figures 1–3**). Chilling stress severely reduced α-amylase activity and total soluble sugars in both rice cultivars by limiting starch degradation depicting that seed reserves were not metabolized under chilling conditions. The ability of plants to degrade starch into soluble sugars reflects their survival and growth rate under a wide range of environments. Our results revealed that seed priming increased the activity of α-amylase, which promoted the hydrolysis of starch into soluble sugars for seed respiration and better growth. Previously, Hussain et al. (2015) also reported that better germination and seedling growth performance of primed rice seedlings were concomitant with enhanced starch metabolism.

In the present study, higher starch metabolism and vigorous seedling growth of rice after seed priming were consistent with the respiration rate (**Figures 1–4**). In non-primed rice seedlings, chilling stress (NP + CS) severely diminished the respiration rate (58–66%) compared with NP + Cn (**Figure 4**). This might be attributed to the excessive production of ROS, which demolished the mitochondrial metabolism leading to reduced respiration rate and energy supply for growing plant tissue (Taylor et al., 2002). Contrarily in primed rice seedlings, higher respiration rate and more ATP production might have triggered the seedling growth of rice under chilling stress. Palmiano and Juliano (1972) suggested that rapid increase of respiration rate triggered the emergence and seedling development. Paul and Mukherji (1972) attributed the rise in respiration rate to the higher activity of α-amylase enzymes, which suggested that the increase in respiration rate of primed seedlings was strongly linked with the activated starch metabolism and the subsequent seedling growth.

All the seed priming treatments expect HP + CS significantly enhanced (23–32%) the accumulation of free proline in rice seedlings with respect to NP + CS (**Figure 5A**). Proline is a small molecule, which is known to function as an osmotic agent as well as a radical scavenger in plants under chilling stress (Hayat et al., 2012). Higher proline content alleviates the stress damage in plant cells by reducing the water potential (Rathinasabapathi, 2000) and by osmotic adjustment in plants (Watanabe et al., 2000). While examining salinity stress in alfalfa, Hu et al. (2006) reported that higher seed germination and seedling growth after seed priming was associated with enhanced soluble sugar and proline concentrations.

Exposure of chilling stress to non-primed rice seedlings (NP + CS) led to higher MDA and H_2_O_2_ content (**Figures 5B,C**). The H_2_O_2_ is one type of ROS, and its higher level in non-primed rice seedlings under chilling stress, indicates the occurrence of oxidative stress. While MDA is an indicator of lipid peroxidation, and reveals the oxidative damages on membranes. Gill and Tuteja (2010) concluded that exposure of plants to unfavorable environmental conditions leads to higher lipid peroxidation due to the generation of ROS. All the seed priming treatments recorded significantly lower MDA and H_2_O_2_ under chilling stress compared with NP + CS (**Figures 5B,C**). The lower MDA and H_2_O_2_ concentration in primed rice seedlings indicates that chilling-induced oxidative stress and seedling damage/chilling injury were effectively alleviated by seed priming. Previously, a positive effect of seed priming in averting lipid peroxidation and ROS generation was also noticed for lucerne (Zhang et al., 2007) and rice (Zheng et al., 2015) seedlings.

The increased level of antioxidant capacity confers the ability of plants to scavenge ROS and to withstand the chilling stress. The protective role of SOD, POD, CAT, and GSH against chilling induced-oxidative stress is well evident in many crop plants (Gill and Tuteja, 2010). In the present study, seed priming treatments triggered the activities of SOD, POD, and CAT and enhanced the GSH content in rice seedlings under chilling stress (**Figure 6**). The enhanced antioxidant activity in primed rice seedlings, corresponding to the better growth may account for their higher ROS scavenging ability and greater tolerance to chilling stress. However, less antioxidant activities in non-primed rice seedlings suggested the inability of these plants to cope with stress-induced oxidative damage. The SOD plays an important role in catalyzing the dismutation of superoxide, while CAT and POD contribute in scavenging of H_2_O_2_ (Anjum et al., 2015; Fahad et al., 2015). Seed priming-induced increases in SOD, POD, and CAT activities of rice seedlings have also been reported by Khaliq et al. (2015) and Zheng et al. (2015). The GSH, a non-enzymatic antioxidant, is involved in many cellular processes under stress, and is a substrate for glutathione S-transferase and glutathione peroxidase (Foyer and Noctor, 2005). In addition, GSH can directly detoxify superoxide and hydroxyl radical and thus contribute to non-enzymatic ROS scavenging (Kao, 2015). Kocsy et al. (2001) reported that the increasing GSH synthesis increased the chilling tolerance of maize, while inhibiting GSH synthesis reduced the chilling tolerance. A positive correlation between tolerance of chilling-induced photo-inhibition and high glutathione activities has been demonstrated in rice (Kuk et al., 2003; Huang and Guo, 2005). It has been reported that respiration plays a key role in the synthesis of GSH. During respiration, various metabolites such as glycine are produced, which might be used for the synthesis of GSH (Noctor et al., 1999). In the present study, higher GSH content (**Figure 6D**) in primed rice seedlings were concomitant with higher respiration rate (**Figure 4**), which suggests that higher respiration rate may protect the plants against oxidative damage under chilling stress.

The results revealed that Se + CS and SA + CS were more effective under chilling stress than all other seed priming treatments in both rice cultivars. The greater germination and seedling growth performance of rice in these two treatments was linked with the greater starch metabolism, high respiration rate, better anti-oxidative defense system and reduced lipid peroxidation than rest of the treatments under chilling stress. Previous researchers have concluded that the positive effects of Se under different abiotic stresses occur presumably due to the protection against oxidative damage, increased starch synthesis, increased phosphorylation and ATP content, and regulation of plant water status (Feng et al., 2013; Khaliq et al., 2015). Moreover, increasing evidences have suggested that benzoic acid derivative s such as SA regulated the stress tolerance in plants. These molecules trigged the expression of the potential to tolerate stress rather than having any direct effect as a protectant. While reviewing a number of studies, Pál et al. (2013) concluded that SA protects the plant from chilling stress by modifying the antioxidant capacity, changing expression rates of certain genes, and by playing a key role in acclimation process.

The results of the present study clarified that the seed priming did not simply accelerate the germination-related processes but was also involved in other specific mechanisms that improved the seedling vigor and allowed the rice seedlings to cope with the chilling stress. Increase in activities of antioxidants as governed by seed priming (**Figure 6**), can protect the degradation of enzymes due to chilling-induced ROS and maintain the integrity of membranes. In addition to providing protection from oxidative stress, seed priming was also involved in regulating the respiration rate and starch metabolism in the rice seedlings, which might also an important manifestation of enhanced vigor and stress tolerance in plants.

## Conclusion

In short, exposure of chilling stress at 18°C severely hampered the germination and seedling growth performance of both rice cultivars, nevertheless, seed priming treatments effectively assuaged the damaging effects of chilling stress. All the seed priming treatments recorded rapid germination and vigorous seedling growth of both rice cultivars under chilling stress. Seed priming with Se or SA, was found to be more effective among all treatments to thrive under chilling stress. The better germination and vigorous growth of primed-rice seedlings was associated with (1) higher starch metabolism, (2) enhanced respiration rate, (3) better membrane integrity (4) higher metabolite synthesis, and (5) increased activities of antioxidants in these seedlings. However, further studies at transcriptomic and proteomic levels are inevitable to explore the molecular mechanisms of seed priming-induced chilling tolerance.

## Author Contributions

SH and LN initiated and designed the research, SH and FK performed the experiments and collected the data, SH, HH, and LN analyzed the data and wrote the manuscript.

## Conflict of Interest Statement

The authors declare that the research was conducted in the absence of any commercial or financial relationships that could be construed as a potential conflict of interest.
